# Effects of Hospice Care on Anxiety, Depression, and Pain in Patients With Terminal Colon Adenocarcinoma: A Retrospective Cohort Study

**DOI:** 10.62641/aep.v54i2.2192

**Published:** 2026-04-15

**Authors:** Xiaoyan Wang, Simeng Chen

**Affiliations:** ^1^Oncology Ward, The Affiliated Yangming Hospital of Ningbo University (Yuyao People’s Hospital of Zhejiang Province), 315400 Yuyao, Zhejiang, China

**Keywords:** colon cancer, depression, anxiety, hospice care, pain, palliative medicine

## Abstract

**Background:**

Terminal colon adenocarcinoma is a debilitating condition often accompanied by severe pain and substantial anxiety and depression. Hospice care provides a dedicated framework to address this symptom complex, yet robust evidence for its real-world effectiveness within the Chinese healthcare context remains underdeveloped and insufficiently documented. This study aimed to evaluate the effects of hospice care on key clinical outcomes in terminal colon adenocarcinoma.

**Methods:**

This retrospective cohort analysis reviewed data from 92 patients with histologically confirmed terminal colon adenocarcinoma (≥stage Ⅲ) treated at The Affiliated Yangming Hospital of Ningbo University between January 2024 and June 2025. The cohort included 46 patients receiving integrated hospice care alongside standard oncology treatment and 46 matched controls receiving standard care only. Comparative analyses of depression (Hospital Anxiety and Depression Scale–Depression, HADS-D), anxiety (HADS-A), pain (visual analogue scale, VAS), opioid usage, and healthcare utilisation were conducted at baseline, 1, 3, and 6 months.

**Results:**

Baseline characteristics were comparable between groups. HADS-D scores decreased more in the hospice care group (from 9.13 ± 3.39 to 5.91 ± 2.72) than in the standard group (from 9.35 ± 3.58 to 8.57 ± 3.04; *p* < 0.001). HADS-A scores showed a greater reduction in the hospice care group (from 8.63 ± 2.55 to 5.83 ± 2.57) than in the standard group (from 9.13 ± 3.17 to 8.48 ± 2.63; *p* < 0.001). The hospice care group demonstrated significantly greater reductions in VAS scores (from 6.83 ± 1.19 to 3.17 ± 1.01) compared with the standard group (from 6.74 ± 1.40 to 5.51 ± 1.63; *p* < 0.001) and a higher proportion achieved ≥30% pain reduction at 6 months (80.43% vs. 39.13%, *p* < 0.001). Additionally, hospice care was associated with lower opioid consumption, shorter hospital stays, fewer emergency visits, and reduced re-admissions (all *p* < 0.05), with no increase in adverse events.

**Conclusions:**

For patients with terminal colon adenocarcinoma, integrated hospice care was associated with significantly improved pain control and reduced anxiety and depressive symptoms. It was also associated with decreased healthcare utilisation with a favourable safety profile.

## Introduction

Colon adenocarcinoma is one of the most common malignant tumours worldwide and remains a leading cause of cancer-related morbidity and mortality [1, 2, 3]. With continuous improvements in diagnostic techniques and treatment strategies, the survival rate of patients with colon cancer has increased remarkably in recent years [4, 5]. Owing to the improved survival rate, a spectrum of physical and psychological challenges has emerged as important concerns both during and after cancer treatment [6]. In particular, cancer-related pain, and emotional disturbances are quite common and are receiving increasing attention in the clinical and psychosocial oncology literature [7, 8, 9, 10].

Pain is one of the most common and distressing symptoms of colon cancer, and it can happen from tumour invasion, surgical trauma, and treatment-related side effects. Persistent pain not only affects patients’ daily functioning but also contributes to psychological problems such as anxiety and depression. A previous study indicated that approximately 60% of cancer patients without negative emotions before treatment may develop anxiety and depression during chemotherapy [11]. Psychological distress, in turn, has been shown to exacerbate pain perception and negatively impact treatment adherence [12, 13]. Previous studies have investigated the physical and psychological experiences of cancer patients. This research has predominantly focused on Western populations or combined data across various cancer types [14]. In addition, previous research has mainly examined either the direct connection between pain and psychological outcomes, or the separate effect on anxiety and depression [15].

With these problems in mind, we aimed to investigate whether there was a relationship among anxiety, depression, and the pain in patients with colon adenocarcinoma. By integrating physical and mental factors into one analysing system, this study provides a better understanding of the factors contributing to anxiety and depression in patients with colon cancer. The findings may contribute to optimising supportive care strategies and promoting holistic management approaches in oncology practice.

## Materials and Methods

### Study Design and Population

This retrospective cohort study was conducted at The Affiliated Yangming Hospital of Ningbo University between 1 January 2024 and 30 June 2025. A total of 92 patients with histologically confirmed primary colon adenocarcinoma were consecutively enrolled from the Departments of General Surgery and Oncology. To ensure baseline comparability between the hospice care group and the standard group, 1:1 propensity score nearest neighbour matching was performed. Propensity scores were estimated using a logistic regression model, with receipt of integrated hospice care as the dependent variable. Matching variables included key demographic characteristics (age, gender, body mass index), disease-related factors, and baseline outcome indicators (depression, anxiety, and pain levels), all of which may have had potential confounding effects on the study results. A caliper width of 0.2 standard deviations was applied during the matching process to ensure comparability between matched pairs, and no replacement was allowed during matching. The study protocol was reviewed and approved by the Ethics Committee of The Affiliated Yangming Hospital of Ningbo University (Approval No. 2025-12-008). All procedures adhered to the ethical standards of the institutional ethics committee and the principles of the Declaration of Helsinki.

### Inclusion and Exclusion Criteria

Inclusion criteria: (1) age ≥18 years; (2) diagnosed with terminal colon adenocarcinoma (≥stage Ⅲ); (3) estimated life expectancy ≤6 months; and (4) sufficient cognitive function to complete self-reported questionnaires. Patients were excluded if they met any of the following criteria: (1) a history of other malignancies within the past five years; (2) severe neurocognitive impairment; (3) the clinical data were incomplete, with key information (such as core clinical measures, therapeutic regimens) missing or unavailable for supplementation; and (4) being in the curative treatment phase (e.g., resectable surgery, radical chemoradiotherapy).

### Hospice Care Services

The hospice care methods for the hospice care group are as follows [16]: (1) Environmental Care: Create a clean and quiet ward environment for patients by placing green plants and ensuring regular ventilation. (2) Dietary Care: Instruct patients to avoid foods that cause bloating or unpleasant odours, and refrain from consuming foods that may lead to diarrhoea or constipation. Medication was provided when necessary. (3) Daily Care: Regularly clean the patient’s skin and trim their nails to maintain personal hygiene and neatness. (4) Pain Management: Provide individualised interventions for patients, such as etiological treatment and the use of pharmacological and non-pharmacological methods. Regular follow-ups were conducted via telephone and WeChat, including video channel and official platform. A pain follow-up log was maintained for timely documentation. (5) Cognitive Care: Share the treatment plan with patients, make decisions collaboratively with them, solicit their feedback, and employ various approaches to help alleviate their suffering. (6) Death Education: Communicate with patients at appropriate time to help alleviate their fear of death and prevent extreme behaviours or emotional outbursts. Simultaneously, assist patients in advance with drafting wills and arranging post-mortem affairs during their final moments. (7) Social Support: Social workers assisted terminally ill patients in completing the “Four Tasks” to achieve the goal of “a good death for the departing and a peaceful farewell for those left behind”.

To monitor the fidelity and adherence to the protocol, we implemented the following measures: (1) Expert audits: Monthly, experts in hospice care conducted spot checks on the care process, reviewing the completeness and standardisation of follow-up records and care documentation. (2) Team coordination: Weekly team meetings were held to discuss feedback and issues arising during the intervention, allowing for timely adjustments and optimisation of the protocol to ensure consistency among all staff. (3) Participation tracking: Patient attendance at in-person and online care sessions was recorded. Additionally, telephone follow-ups were conducted monthly to verify the execution status of care from the patient’s perspective.

### Data Collection

Patients’ clinical data, including age, sex, tumour anatomical location, histopathological stage, treatment regimen, comorbid diseases, and questionnaire results, were extracted from the hospital’s medical record system.

To ensure the authenticity, completeness, and accuracy of the research data, a rigorous quality control system was established for this study: (1) All data collectors underwent systematic training to familiarise themselves with the data collection standards, procedures, and relevant terminology for this study. They were required to clearly understand the key points for extracting various types of information; (2) A dual verification mechanism was implemented. Two researchers independently extracted data for the same patient and cross-checked the results. In case of discrepancies, a third researcher reviewed the original medical records for verification to reach a consensus; (3) Collected data were systematically organised and reviewed. For missing critical information, efforts were made to supplement it by reviewing additional medical records or contacting patients or their families for follow-up. If supplementation was not possible, the reasons for the missing data were documented in detail; (4) All collected data were uniformly entered into an Excel database to establish a standardised data archive. Data validation rules were set during entry to prevent input errors. After entry, a dedicated person conducted data verification to ensure the data quality met the requirements for research analysis.

### Outcome Measurement

Several standardised and validated self-report instruments were used. All scales were internationally recognised and psychometrically validated for Chinese populations.

(1) Primary Outcome: Anxiety and depression were assessed using the Hospital Anxiety and Depression Scale (HADS), introduced by Zigmond and Snaith in 1983 [17]. HADS is the scale that is divided equally in 14 items for HADS-A (anxiety) and HADS-D (depression), then being scored on 4-point Likert scale with a range of 0–3, with a higher score indicates higher emotional distress [17]. The Chinese version of HADS has been performed a psychometric study of HADS within the Chinese hospital and community samples. The Cronbach’s α coefficients were 0.75–0.82, indicating fair internal consistency [18, 19].

(2) Secondary Outcomes: i. Pain intensity was assessed using the visual analogue scale (VAS). VAS is a 10 cm long horizontal line with left end as “No pain”, the right end as “Worst pain imaginable”. Patients were asked to mark their pain intensity at the time of assessment on the line [20]. Chinese expert consensus and validation studies support its use for pain assessment in Chinese patients [21, 22]. In the present study the VAS demonstrated excellent test–retest reliability. Pain relief was defined as a reduction in VAS score of ≥3 cm from baseline [23]. ii. Opioid consumption: Measured as daily morphine equivalent dosage (mg/day) during the study period. iii. Healthcare utilisation: Including hospital stay per admission, number of emergency department visits over 6 months, and readmission rate. iv. Adverse events. v. Karnofsky Performance Score (KPS) were used to assesses patients’ performance on a scale from 0 to 100% based on their capacity to carry out daily activities, work, need for assistance, and the presence of disease-related symptoms [24].

All questionnaires completion required approximately 20–25 minutes for each participant, and all responses were reviewed on-site for completeness by trained researchers prior to data entry.

### Statistical Analysis

Statistical analyses were performed using IBM SPSS 26.0 (IBM Corp, Armonk, NY, USA). The normality of continuous variables was assessed using the Shapiro–Wilk test. Normally distributed variables were presented as mean ± standard deviation (SD), while non-normally distributed variables were presented as median (interquartile range) [M (IQR)]. Categorical variables were presented as frequencies and percentages. Comparison between the two groups utilised independent-sample *t*-tests for normally distributed data and the Mann–Whitney U test for non-normally distributed data. Categorical variables were compared using the chi-square test. For the propensity score matching process, balance checking was conducted after matching: standardised mean differences (SMD) were calculated for all matched variables, with SMD <0.1 indicating effective balance of baseline characteristics between groups. All tests were two-tailed, and a *p*-value < 0.05 was considered statistically significant.

## Results

### Baseline Characteristics

The initial screening process evaluated 100 patients for study eligibility. Following application of the exclusion criteria, eight individuals were removed from the patient pool. A total of 92 subjects were finally included after 1:1 propensity score nearest neighbour matching, comprising 46 patients in the hospice care group (who received both standard treatment and hospice care) and 46 patients in the standard group (who received only standard treatment). No significant differences were found in baseline demographic characteristics and clinical parameters between the two groups (all *p* > 0.05). The SMDs for all matched variables were <0.1, confirming effective balance of baseline characteristics. The baseline characteristics is presented in Table 1.

**Table 1.** **Baseline demographic and clinical characteristics**.

Variable	Hospice Care Group (n = 46)	Standard Group (n = 46)	Statistic (*t*/χ^2^)	*p*	SMD
Age (years, mean ± SD)	64.22 ± 9.47	65.13 ± 8.69	*t* = 0.480	0.632	0.098
Gender (Male, n [%])	28 (60.87%)	26 (56.52%)	χ^2^ = 0.179	0.672	0.089
BMI (kg/m^2^)	23.70 ± 3.23	24.13 ± 3.48	*t* = 0.614	0.541	0.125
Disease stage (Stage IV, n [%])	42 (91.30%)	43 (93.48%)	χ^2^< 0.001	1.000	0.076
HADS-D Score	9.13 ± 3.39	9.35 ± 3.58	*t* = 0.303	0.763	0.062
VAS Score	6.83 ± 1.19	6.74 ± 1.40	*t* = 0.332	0.741	0.071
HADS-A Score	8.63 ± 2.55	9.00 ± 3.22	*t* = 0.397	0.692	0.085
Karnofsky Performance Score	62.54 ± 7.79	63.13 ± 8.06	*t* = 0.357	0.722	0.075

Note: BMI, body mass index; VAS, visual analogue scale; HADS-D, Hospital Anxiety and Depression Scale–Depression subscale; HADS-A, Hospital Anxiety and Depression Scale–Anxiety subscale; SMD, standardised mean differences.

### Anxiety and Depressive Symptoms

The hospice care group showed significantly greater improvement in depressive and anxiety symptoms compared with baseline. The between-group difference was most pronounced at the 6-month follow-up for depressive symptoms (5.91 ± 2.72 vs. 8.57 ± 3.04, Δ = –2.66, *p* < 0.001; Table 2) and anxiety symptoms (5.83 ± 2.57 vs. 8.48 ± 2.63, Δ = –2.65, *p* < 0.001; Table 3).

**Table 2.** **Changes in depression scores over time (HADS-D)**.

Time Point	Hospice Care Group	Intra-group Comparison	*p*	Standard Group	Between-group	*p*	Intra-group Comparison	*p*
	(mean ± SD)	vs. Baseline (Δ)		(mean ± SD)	Difference (Δ)		vs. Baseline (Δ)	
Baseline	9.13 ± 3.39	-	-	9.35 ± 3.58	–0.22	0.763	-	-
1 Month	7.19 ± 3.12	1.657	0.016	8.94 ± 3.51	–1.75	0.013	0.160	0.802
3 Months	6.30 ± 2.91	2.327	<0.001	8.79 ± 3.22	–2.49	<0.001	0.308	0.962
6 Months	5.91 ± 2.72	2.783	<0.001	8.57 ± 3.04	–2.66	<0.001	0.459	0.474
*F*	6.412			0.357				
*p*	<0.001			0.784				

Note: Data are presented as mean ± standard deviation. The HADS-D subscale was used to assess depressive symptoms. The *p*-values represent the results of independent-sample *t*-tests comparing the hospice care group and the standard group at each specific time point. Δ denotes the mean difference from baseline (within-group) or between groups. Abbreviations: HADS-D, Hospital Anxiety and Depression Scale–Depression subscale; SD, standard deviation.

**Table 3.** **Changes in anxiety scores over time (HADS-A)**.

Time Point	Hospice Care Group	Intra-group Comparison	*p*	Standard Group	Between-group	*p*	Intra-group Comparison	*p*
	(mean ± SD)	vs. Baseline (Δ)		(mean ± SD)	Difference (Δ)		vs. Baseline (Δ)	
Baseline	8.63 ± 2.55	-	-	9.13 ± 3.17	–0.50	0.407	-	-
1 Month	7.09 ± 3.33	1.457	0.015	9.09 ± 3.41	–2.00	0.006	0.130	0.843
3 Months	6.13 ± 2.68	2.543	<0.001	8.65 ± 3.41	–2.52	<0.001	0.478	0.467
6 Months	5.83 ± 2.57	2.935	<0.001	8.48 ± 2.63	–2.65	<0.001	0.674	0.306
*F*	9.79			0.447				
*p*	<0.001			0.720				

Note: Data are presented as mean ± standard deviation. The HADS-A subscale was used to assess anxiety symptoms. The *p*-values represent the results of independent-sample *t*-tests comparing the hospice care group and the standard group at each specific time point. Δ denotes the mean difference from baseline (within-group) or between groups. Abbreviations: HADS-A, Hospital Anxiety and Depression Scale–Anxiety subscale; SD, standard deviation.

Furthermore, the proportion of patients with clinically significant depression (HADS-D ≥11) decreased significantly in the hospice care group, whereas it remained high in the standard group (χ^2^ = 7.794, *p* = 0.005, Table 4). The proportion of patients with clinically significant anxiety (HADS-A ≥11) decreased significantly in the hospice care group, whereas it remained high in the standard group (χ^2^ = 8.364, *p* = 0.004, Table 5).

**Table 4.** **Changes in the proportion of clinically significant depression**.

Time Point	Hospice Care Group [n (%)]	Standard Group [n (%)]	χ ^2^	*p*
Baseline	24 (52.17%)	25 (54.35%)	0.044	0.834
1 Month	18 (39.13%)	23 (50.00%)	1.100	0.294
3 Months	14 (30.43%)	25 (54.35%)	5.386	0.020
6 Months	11 (23.91%)	24 (52.17%)	7.794	0.005

Note: HADS-D, Hospital Anxiety and Depression Scale–Depression subscale; Clinically significant depression was defined as a HADS-D score ≥11.

**Table 5.** **Changes in the proportion of clinically significant anxiety**.

Time Point	Hospice Care Group [n (%)]	Standard Group [n (%)]	χ ^2^	*p*
Baseline	9 (19.57%)	16 (34.78%)	2.691	0.101
1 Month	8 (17.39%)	17 (36.96%)	4.449	0.035
3 Months	5 (10.87%)	13 (28.26%)	4.420	0.036
6 Months	1 (2.17%)	10 (21.74%)	8.364	0.004

Note: HADS-A, Hospital Anxiety and Depression Scale–Anxiety subscale; Clinically significant anxiety was defined as a HADS-A score ≥11.

### Pain Intensity

As shown in Table 6, baseline VAS scores were comparable between the hospice care and standard groups (*p* > 0.05). The hospice care group demonstrated significantly greater reductions in VAS scores at all follow-up time points (all *p* < 0.001), with scores decreasing to 3.17 ± 1.01 at 6 months, compared with 5.51 ± 1.63 in the standard group. As shown in Table 7, a significantly higher proportion of patients in the hospice care group achieved clinically meaningful pain relief at each time point. At 6 months, 80.43% in the hospice care group met this criterion, versus 39.13% in the standard group (χ^2^ = 16.320, *p* < 0.001).

**Table 6.** **Changes in VAS scores over time**.

Time Point	Hospice Care Group	Intra-group Comparison	*p*	Standard Group	Between-group	*p*	Intra-group Comparison	*p*
	(mean ± SD)	vs. Baseline (Δ)		(mean ± SD)	Difference (Δ)		vs. Baseline (Δ)	
Baseline	6.83 ± 1.19	-	-	6.74 ± 1.40	0.09	0.741	-	-
1 Month	4.52 ± 1.03	2.370	<0.001	6.01 ± 1.27	–1.49	<0.001	1.002	0.001
3 Months	3.83 ± 1.06	3.099	<0.001	5.79 ± 1.53	–1.96	<0.001	1.156	<0.001
6 Months	3.17 ± 1.01	3.444	<0.001	5.51 ± 1.63	–2.34	<0.001	1.258	<0.001
*F*	93.813			7.306				
*p*	<0.001			<0.001				

Note: Data are presented as mean ± standard deviation. Pain intensity was assessed using the Visual Analogue Scale (VAS). The *p*-values represent the results of independent-sample *t*-tests comparing the hospice care group and the standard group at each specific time point. Δ denotes the mean difference from baseline (within-group) or between groups.

**Table 7.** **Pain relief rate comparison**.

Time Point	Hospice Care Group [n (%)]	Standard Group [n (%)]	χ ^2^	*p*
1 Month	31 (67.39%)	15 (32.61%)	11.130	0.001
3 Months	35 (76.09%)	17 (36.96%)	14.331	<0.001
6 Months	37 (80.43%)	18 (39.13%)	16.320	<0.001

Note: Pain intensity was assessed using the Visual Analogue Scale (VAS); Pain relief was defined as a reduction in VAS score of ≥3 cm from baseline.

### Other Secondary Outcomes

The hospice care group had significantly lower daily opioid consumption (*p* < 0.001), shorter average hospital stays per admission (*p* = 0.003), and fewer emergency department visits over six months (*p* < 0.001) compared with the standard group. The readmission rate was also lower in the hospice care group (15.22% vs. 34.78%, *p* = 0.030, Table 8).

**Table 8.** **Comparison of secondary outcomes**.

Variable	Hospice Care Group (n = 46)	Standard Group (n = 46)	Statistic (*t*/χ^2^)	*p*
Opioid consumption (morphine equivalent, mg/day)	72.35 ± 18.40	89.68 ± 20.10	*t* = 4.313	<0.001
Hospital stays (days per admission)	6.09 ± 3.20	8.43 ± 4.01	*t* = 3.094	0.003
Emergency visits (6 months)	1.22 ± 0.79	2.02 ± 1.12	*t* = 3.959	<0.001
Readmission rate (%)	7 (15.22%)	16 (34.78%)	χ^2^ = 4.696	0.030

Note: Data are presented as mean ± standard deviation or n (%). Between-group comparisons were performed using independent-sample *t*-tests for continuous variables and a chi-square test for the readmission rate.

### Safety Analysis

The incidence of adverse events, including sedation, respiratory depression, falls, and infections, was low and did not differ significantly between the two groups (all *p* > 0.05, Table 9). No serious or unexpected treatment-related adverse events were reported during the study.

**Table 9.** **Incidence of adverse events**.

Adverse Event Type	Hospice Care Group n (%)	Standard Group n (%)	χ ^2^	*p*
Sedation/Altered consciousness	3 (6.52%)	5 (10.87%)	0.137	0.711
Respiratory depression	1 (2.17%)	2 (4.35%)	<0.001	1.000
Falls	2 (4.35%)	3 (6.52%)	<0.001	1.000
Infection	5 (10.87%)	6 (13.04%)	0.103	0.748
Others	2 (4.35%)	2 (4.35%)	0.261	0.609

Note: Data are presented as n (%). Between-group comparisons of the incidence of each adverse event were performed using chi-square tests.

## Discussion

Colon adenocarcinoma remains a major public health concern worldwide, and with increasing survival time, the need for comprehensive end-of-life care has become more urgent [25]. For patients with terminal colon adenocarcinoma, their condition is characterised by chronic pain, severe anxiety, and a progressively declining quality of life. Hospice care pays attention to overall physical, emotional, and social support, is increasingly seen as necessary.

The present study demonstrated that hospice care significantly reduced anxiety and depressive symptoms and alleviated pain in patients with terminal colon adenocarcinoma. The hospice care group had lower opioid consumption, fewer emergency department visits, and lower readmission rates than the standard care group, while demonstrating comparable safety outcomes. These results give strong support for hospice care as an important part of the management of terminal cancer patients.

Depression and anxiety are common comorbidities in cancer patients [26]. About 7.9%–32.4% of patients experience depression after diagnosis [27]. Cancer patients with depression or anxiety tend to have more severe physical and psychological symptoms and have poorer prognosis [28]. Psychological distress adversely affects treatment compliance and overall well-being. The prevalence of anxiety and depression in patients with colon adenocarcinoma ranges from approximately 1.0% to 47.2% and 1.6% to 57%, respectively [29]. A study has shown that, compared with the general population, patients with colon adenocarcinoma have a significantly higher risk of depression even five years after diagnosis [30]. A recent Canadian study, which stratified participants by gender, also found a higher risk of depression in male patients with colon adenocarcinoma than in those without cancer [31]. Inflammatory mediators, such as C-reactive protein, together with a variety of biological processes, tissue damage, and chronic stress responses, may predispose cancer patients to depression [32]. The present study observed substantial reductions in depressive symptoms within the hospice cohort, as evidenced by significantly decreased HADS-D scores at the six-month follow-up. These findings align with existing researches documenting the efficacy of meaning-cantered psychotherapy in palliative settings for addressing both depressive manifestations and existential suffering [33, 34]. The therapeutic benefits may be mediated through systematic emotional support, consistent psychological dialogue, and specialised affective care provided by hospice teams. These interventions collectively facilitate stress adaptation while fostering dignity and acceptance during terminal illness, thereby ameliorating depressive symptoms.

We also found that the hospice care group had less pain than the standard care group, which is in accordance with former studies demonstrating that comprehensive palliative care provides great pain control in advanced cancer patients [35, 36]. Specific interventions of hospice care include structured relaxation techniques and specialised psychological support services. These elements collectively establish an optimised framework for comprehensive pain control [37]. The modification of central pain processing mechanisms represents another potential benefit. Enhanced emotional stability and diminished anxiety levels are frequently observed in patients receiving hospice care [16]. Additionally, reduced pain intensity (a secondary outcome) may directly alleviate depressive symptoms, as pain and depression are positively associated in our study. Pain is associated with depression through several potential mechanisms. First, pain may limit physical activity [38], and inactivity is a known risk factor for depression in adults [39]. Second, pain is often accompanied by reduced mobility and functional decline, both of which are significant contributors to depression [40]. Additionally, pain can lead to social isolation and loneliness, thereby fostering depressive symptoms. For example, the English Longitudinal Study of Ageing found that moderate-to-severe pain at baseline was associated with loneliness four years later [41]. Similarly, another study had indicated a link between pain and loneliness in older adults [42].

The investigation documented further advantages of hospice care beyond clinical and psychological dimensions. Hospice care can directly improve outcomes and support better and more appropriate use of healthcare services among people with advanced cancer [43]. This systematic approach effectively minimises the occurrence of acute symptom deterioration and consequent urgent medical service utilisation [44, 45].

Several methodological limitations warrant consideration. The current study was conducted at a single tertiary medical centre, resulting in a dataset with relatively limited scope, which may restrict the generalisability of these findings to broader populations. Furthermore, potential subjective bias inherent in patient-reported outcomes persists as a methodological consideration. Because this was an open-label study, especially outcomes like pain are based on patient reports and may therefore be subject to reporting bias. Third, six months of follow-up might be too short to determine the long-term sustainability of the benefits associated with hospice care. Finally, some potential confounders like social support and coping skills, were not captured and could be having an impact.

## Conclusions

This study provides robust evidence that integrated hospice care is safe and effective for patients with terminal colon adenocarcinoma. Compared with standard oncology care alone, hospice care delivers superior pain relief, alleviates anxiety and depression, reduces opioid consumption, shortens hospital stays, and decreases emergency visits and readmissions without raising adverse events. These benefits confirm that hospice care addresses both physical and psychological distress, enhances patient comfort and dignity, and optimizes healthcare resource use at the end of life. We recommend routine incorporation of hospice care into clinical pathways for advanced colon cancer. Future multi-center, long-term studies are warranted to validate these findings and explore cost-effectiveness and generalizability across diverse healthcare settings.

## Availability of Data and Materials

All experimental data included in this study can be obtained by contacting the corresponding author if needed.
